# Supplementary material for: Hairy root transformation system as a tool for CRISPR/Cas9-directed genome editing in oilseed rape (Brassica napus)
Source: Front Plant Sci. 2022 Aug 4;13:919290. doi: 10.3389/fpls.2022.919290 (PMC9386449; doi:10.3389/fpls.2022.919290)
Supplement: SUPPLEMENTARY FIGURE 1 — Phylogenetic analysis of TAA1, TAR1, and TAR2 proteins in Brassica napus and related species. [file Data_Sheet_1.PDF]

Supplementary Material

Hairy root transformation system as a tool for CRISPR/Cas9-directed genome editing in oilseed rape (*Brassica napus*)

Supplementary Table S1. List of oligonucleotides

Supplementary Table S2. Plasmid construction for CRISPR/Cas9-mediated genome editing using MoClo system

Supplementary Table S3. Origin of plasmids

Supplementary Table S4. Mutagenesis efficiency of each construct for each gRNA loci

Supplementary Table S5. Stability of homozygous loci in hairy root regenerants

Supplementary Figure S1. Phylogenetic analysis of TAA1, TAR1, and TAR2 proteins in *Brassica napus* and related species

Supplementary Figure S2. Hairy roots induction in different *B. napus* cultivars

Supplementary Figure S3. Optimization of hairy roots regeneration in *B. napus* DH12075

Supplementary Figure S4. Phenotypes of *BnaTAA1* double mutant primary inflorescence compared to the one of wild-type DH12075 plants

Supplementary Figure S5. Workflow scheme for transgene-free *BnaTAA1*-edited plants generation

Supplementary Table S1. List of oligonucleotides.

Guides

(Esp31 restriction sites in red lower case, cut site in red upper case, guide sequence in green)

BnaTAA1_SaGuide1_fw	GTGcgtctcAATTGGCCGTCGGAAACGCAGCAACCGTTTGgagacgCAC
BnaTAA1_SaGuide1_rev	GTGcgtctcCAAACGGTTGCTGCGTTTCCGACGGCCAATTgagacgCAC
BnaTAA1_SaGuide2_fw	GTGcgtctcAATTGGGCCAGTAATAAGCAAAGTCGTTTGgagacgCAC
BnaTAA1_SaGuide2_rev	GTGcgtctcCAAACGACTTTGCTTATTACTGGCCCAATTgagacgCAC
BnaTAA1_pcoGuide1_fw	GTGcgtctcAATTGGAAGCGATCAAAGAGTTGCAGTTTGgagacgCAC
BnaTAA1_pcoGuide1_rev	GTGcgtctcCAAACTGCAACTCTTTGATCGCTTCCAATTgagacgCAC
BnaTAA1_pcoGuide2_fw	GTGcgtctcAATTGGACGTCACCAGCTCGATGAAGTTTGgagacgCAC
BnaTAA1_pcoGuide2_rev	GTGcgtctcCAAACTTCATCGAGCTGGTGACGTCCAATTgagacgCAC

BnaAnng22030D cloning

BnaAnng22030D_fw	ATGGTGAAACTGGAGAACATGAAG
BnaAnng22030D_rev	TGTTAATGGTATTTAGTTTGGTTCTACATC

BnaTAA1 genes amplification and discrimination of BnaA02.TAA1 and BnaC02.TAA1

BnaC02g19980D_amp_fw	GGTTGGTGAACGTAGAGGTGA
BnaC02g19980D_amp_rev	TTTTTCGTCAGGTCATCTAGTATATTT
BnaA02g14990D_amp_fw	ACCAAGAAGAAGAAGACGATGG
BnaA02g14990D_amp_rev	GACAGACACCGAGAGAGAACCT

Colony PCR with selected bacterial clones

SaCas9_detect_fw	CAACCTCCTCACCGATCACT
SaCas9_detect_rev	AAGCTCTGCCACGTACTTCT
pcoCas9_detect_fw	AAGTACTTGAAAGCAGCTG
pcoCas9__detect_rev	GAACCGCTCTTATCAAGAAG
RNAscaffold_detect_fw	TCTTGAACCGTAGCTTTCGT
RNAscaffold_detect_rev	ATGGGCCTGCTTCTCTTCTT

Genotyping of hairy roots regenerants and T1 plants

SaCas9_detect_fw	CAACCTCCTCACCGATCACT
SaCas9_detect_rev	AAGCTCTGCCACGTACTTCT
pcoCas9_detect_fw	AAGTACTTGAAAGCAGCTG
pcoCas9__detect_rev	GAACCGCTCTTATCAAGAAG
TL_rolA_fw	GTTAGGCGTGCAAAGGCCAAG
TL_rolA_rev	TGCGTATTAATCCCGTAGGTC
TL_rolB_fw	AAAGAAGGTGCAAGCTACCTCTCT
TL_rolB_rev	AAAGTCTGCTATCATCCTCCTATG
TL_rolC_fw	TACGTCGACTGCCCGACGATGATG
TL_rolC_rev	AAACTTGCACTCGCCATGCCTCAC
TR_aux1_fw	CATAGGATCGCCTCACAGGT
TR_aux1_rev	CGTTGCTTGATGTCAGGAGA
TR_aux2_fw	AACGATAATAGCCCGCTGTG
TR_aux2_rev	CGTCTTGGGTTTGTGGTTCT
TR_mas1_fw	ACCTTGGTACTGCCCAGCCAC
TR_mas1_rev	CTTCAGTGGTCCATACCCACC
TR_ags1_fw	GGTGTTACGTCCTTGATGTGTTCTGC
TR_ags1_rev	ATGCGCGTCAACGATTCGCCCT
virC_fw	AATGCGTCTCTCTCGTGCAT
virC_rev	AAACCGACCACTAACGCGAT

3´and 5´RACE (nested PCR)

3-TAA_fw_outer	TTATCTTTGCTTGCTGGTGG
3-TAA_fw_inner	GTTTCAATAAGAAGGGTCCGTT
5-TAA_rev_outer	AATCACTTTCCTTCACCACTTCACGAAG
5-TAA_rev_inner	TCTCCTTGAGAACTTTGAGAATCTTG

Alternative splicing analysis

TAA_cds_fw	TTACACACTAACAAAACATAAGAGCAA
TAA_cds_rev	AACCATTAGACTTTTATATGCGCTTATTA

RT-qPCR

Q_TAA_fw	CAAGACACTCGAATCTTACCCTGCGTT
Q_TAA_rev	CTCCTGCCCTGCTCATAACCTTCT
Q_TBP2_fw	TGGCTTTCCTGCAAAGTTCAAGGATTTC
Q_TBP2_rev	GTGTAAGTCTCTTCTCTCATCTTGGCTCC

Off-target analysis

Off_chrA03_fw	CAATGTTTCATTGGATATGCCCAGAGT
Off_chrA03_rev	GTTAGGACTGTTGAGACTTTGTGGGA
Off_chrA09_fw	TGAGTGATGAAAGAAAGCTGGATTACA
Off_chrA09_rev	ACATTGAAACCCTTGTAGCCTTGTATT
Off_chrA10_fw	TGTTAATTTTATTGATCATGCGACTTATG
Off_chrA10_rev	GTCAAATGTAAGCGTCGTGCAACAC
Off_chrAnn_random_fw	TTCCGTCATCAATCTGGATCAGTACGTT
Off_chrAnn_random_rev	CTACTAGTAAAAAGACCTTAGTTATATGGC
Off_chrC03_fw	GGGCAGTTTTTATACGCTTTAGGC
Off_chrC03_rev	AGTGATGGTGAGAAACACTGCACAC
Off_chrC05_fw	TTCATCACTCGTTCTCTCTACATCCG
Off_chrC05_rev	CCTTACTCGCAGTACACTGTCGAG
Off_chrC06_random_fw	TTCCGTCATCAATCTGGATCAGTACGTT
Off_chrC06_random_rev	CCACGAAATATGGATTCGCGATTGGTT

Supplementary Table S2. Plasmid construction for CRISPR/Cas9-mediated genome editing using Moclo system.

Cas9 cassette cloning

Name	Backbone	Promotor+5´UTR	CDS	Terminator
pL1M-R2-pCaMV35SShort-pcoCas9-t35S	pICH47811 pL1V-R2	pICH51277 pL0M-P-CaMV35SShort_5UTMV	pcoCas9-gene	pICH41414 pL0M-T-35S
pL1M-R2-pCaMV35SLong-pcoCas9-t35S	pICH47811 pL1V-R2	pICH51266 pL0M-P-CaMV35SLong_5UTMV	pcoCas9-gene	pICH41414 pL0M-T-35S
pL1M-R2-pCaMV35SShort-SaCas9-t35S	pICH47811 pL1V-R2	pICH51277 pL0M-P-CaMV35SShort_5UTMV	SaCas9-gene	pICH41414 pL0M-T-35S
pL1M-R2-pCaMV35SLong-SaCas9-t35S	pICH47811 pL1V-R2	pICH51266 pL0M-P-CaMV35SLong_5UTMV	SaCas9-gene	pICH41414 pL0M-T-35S

Name	Backbone	Promotor	5-UTR	CDS	Terminator
pL1M-R2-pCaMV35SShort-NLS-pcoCas9-t35S	pICH47811 pL1V-R2	pICH41388 pL0M-P-CaMV35SShort	pAGM5331 pL0M-5U-TMV_SP-SV40	pcoCas9-gene	pICH41414 pL0M-T-35S
pL1M-R2-pCaMV35SLong-NLS-pcoCas9-t35S	pICH47811 pL1V-R2	pICH41373 pL0M-P-CaMV35SLong	pAGM5331 pL0M-5U-TMV_SP-SV40	pcoCas9-gene	pICH41414 pL0M-T-35S
pL1M-R2-pCaMV35SShort-NLS-SaCas9-t35S	pICH47811 pL1V-R2	pICH41388 pL0M-P-CaMV35SShort	pAGM5331 pL0M-5U-TMV_SP-SV40	SaCas9-gene	pICH41414 pL0M-T-35S
pL1M-R2-pCaMV35SLong-NLS-SaCas9-t35S	pICH47811 pL1V-R2	pICH41373 pL0M-P-CaMV35SLong	pAGM5331 pL0M-5U-TMV_SP-SV40	SaCas9-gene	pICH41414 pL0M-T-35S
pL1M-R2-pRbcS2B-NLS-SaCas9-t35S	pICH47811 pL1V-R2	pICH44157-P-RbcS2B	pAGM5331 pL0M-5U-TMV_SP-SV40	SaCas9-gene	pICH41414 pL0M-T-35S

GFP cassette cloning

Name	Backbone	Promotor+5´UTR	CDS	Terminator
pL1M-R3-pCaMV35SShort-GFP-t35S	pICH47822 pL1V-R3	pICH51277 pL0M-P-CaMV35SShort_5UTMV	pICH41531 pL0M-SC-GFP	pICH41414 pL0M-T-35S
pL1M-R3-pCaMV35SDouble-GFP-t35S	pICH47822 pL1V-R3	pICH51288 pL0M-P-CaMV35SDouble_5UTMV	pICH41531 pL0M-SC-GFP	pICH41414 pL0M-T-35S
pL1M-R3-pCaMV35SLong-GFP-t35S	pICH47822 pL1V-R3	pICH51266 pL0M-P-CaMV35SLong_5UTMV	pICH41531 pL0M-SC-GFP	pICH41414 pL0M-T-35S

sgRNA cassette cloning (scaffold only)

Name	Backbone	gene/empty sgRNA
pL1M-F4-pAtU6-LacZ-pcoCas9-RNA-scaffold	pICH47761 pL1V-F4	pAtU6-26-LacZ-pcoCas9-RNA-scaffold
pL1M-F5-pAtU6-LacZ-pcoCas9-RNA-scaffold	pICH47772 pL1V-F5	pAtU6-26-LacZ-pcoCas9-RNA-scaffold

pL1M-F4-pAtU6-LacZ-SaCas9-RNA-scaffold	pICH47761 pL1V-F4	pAtU6-26-LacZ-SaCas9-RNA-scaffold
pL1M-F5-pAtU6-LacZ-SaCas9-RNA-scaffold	pICH47772 pL1V-F5	pAtU6-26-LacZ-SaCas9-RNA-scaffold

sgRNA cassette cloning (with guides)

Name	Backbone	Oligonucleotide	Oligonucleotide
pL1M-F4-pAtU6-pcoGuide1-pcoCas9-RNA-scaffold	pL1M-F4-pAtU6-LacZ-pcoCas9-RNA-scaffold	BnTAA1_pcoGuide1_fw	BnTAA1_pcoGuide1_rev
pL1M-F5-pAtU6-pcoGuide2-pcoCas9-RNA-scaffold	pL1M-F5-pAtU6-LacZ-pcoCas9-RNA-scaffold	BnTAA1_pcoGuide2_fw	BnTAA1_pcoGuide2_rev
pL1M-F4-pAtU6-SaGuide1-SaCas9-RNA-scaffold	pL1M-F4-pAtU6-LacZ-SaCas9-RNA-scaffold	BnTAA1_SaGuide1_fw	BnTAA1_SaGuide1_rev
pL1M-F5-pAtU6-SaGuide2-SaCas9-RNA-scaffold	pL1M-F5-pAtU6-LacZ-SaCas9-RNA-scaffold	BnTAA1_SaGuide2_fw	BnTAA1_SaGuide2_rev

L2 vectors

Name	Backbone	Position 1	Position 2	Position 3	End linker
pL2B-KAN-Short35S:pcoCas9-empty	pAGM4673 pL2V	R1-pNOS-KAN-tNOS	pL1M-R2-pCaMV35SShort-pcoCas9-t35S	pL1M-R3-pCaMV35SShort-GFP-t35S	pICH49277 pL1M-ELB-3
pL2B-KAN-Long35S:pcoCas9-empty	pAGM4673 pL2V	R1-pNOS-KAN-tNOS	pL1M-R2-pCaMV35SLong-pcoCas9-t35S	pL1M-R3-pCaMV35SLong-GFP-t35S	pICH49277 pL1M-ELB-3
pL2B-KAN-Short35S:SaCas9-empty	pAGM4673 pL2V	R1-pNOS-KAN-tNOS	pL1M-R2-pCaMV35SShort-SaCas9-t35S	pL1M-R3-pCaMV35SShort-GFP-t35S	pICH49277 pL1M-ELB-3
pL2B-KAN-Long35S:SaCas9-empty	pAGM4673 pL2V	R1-pNOS-KAN-tNOS	pL1M-R2-pCaMV35SLong-SaCas9-t35S	pL1M-R3-pCaMV35SLong-GFP-t35S	pICH49277 pL1M-ELB-3

L2 vectors

Name	Backbone	Position 1	Position 2	Position 3	End linker
pL2B-KAN-Short35S:NLS: pcoCas9-empty	pAGM4673 pL2V	R1-pNOS-KAN-tNOS	pL1M-R2-pCaMV35SShort-NLS-pcoCas9-t35S	pL1M-R3-pCaMV35S Short-GFP-t35S	pICH49277 pL1M-ELB-3
pL2B-KAN-Long35S:NLS: pcoCas9-empty	pAGM4673 pL2V	R1-pNOS-KAN-tNOS	pL1M-R2-pCaMV35SLong-NLS-pcoCas9-t35S	pL1M-R3-pCaMV35S Long-GFP-t35S	pICH49277 pL1M-ELB-3
pL2B-KAN-Short35S:NLS: SaCas9-empty	pAGM4673 pL2V	R1-pNOS-KAN-tNOS	pL1M-R2-pCaMV35SShort-NLS-SaCas9-t35S	pL1M-R3-pCaMV35S Short-GFP-t35S	pICH49277 pL1M-ELB-3
pL2B-KAN-Long35S:NLS: SaCas9-empty	pAGM4673 pL2V	R1-pNOS-KAN-tNOS	pL1M-R2-pCaMV35SLong-NLS-SaCas9-t35S	pL1M-R3-pCaMV35S Long-GFP-t35S	pICH49277 pL1M-ELB-3
pL2B-KAN-RbcS2B:NLS: SaCas9-empty	pAGM4673 pL2V	R1-pNOS-KAN-tNOS	pL1M-R2-pRbcS2B-NLS-SaCas9-t35S	pL1M-R3-pCaMV35S Double-GFP-t35S	pICH49277 pL1M-ELB-3

L2 vectors (Cas9 + guides)

Name	Backbone	Position 4	Position 5	End linker
pL2B-KAN-Short35S:pcoCas9	pL2B-KAN-Short35S: pcoCas9-empty	pL1M-F4-pAtU6-pcoGuide1-pcoCas9-RNA-scaffold	pL1M-F5-pAtU6-pcoGuide2-pcoCas9-RNA-scaffold	pICH41800 pL1M-ELE-5
pL2B-KAN-Long35S:pcoCas9	pL2B-KAN-Long35S: pcoCas9-empty	pL1M-F4-pAtU6-pcoGuide1-pcoCas9-RNA-scaffold	pL1M-F5-pAtU6-pcoGuide2-pcoCas9-RNA-scaffold	pICH41800 pL1M-ELE-5
pL2B-KAN-Short35S:SaCas9	pL2B-KAN-Short35S: SaCas9-empty	pL1M-F4-pAtU6-SaGuide1-SaCas9-RNA-scaffold	pL1M-F5-pAtU6-SaGuide2-SaCas9-RNA-scaffold	pICH41800 pL1M-ELE-5
pL2B-KAN-Long35S:SaCas9	pL2B-KAN-Long35S: SaCas9-empty	pL1M-F4-pAtU6-SaGuide1-SaCas9-RNA-scaffold	pL1M-F5-pAtU6-SaGuide2-SaCas9-RNA-scaffold	pICH41800 pL1M-ELE-5
pL2B-KAN-Short35S:NLS:pcoCas9	pL2B-KAN-Short35S: NLS:pcoCas9-empty	pL1M-F4-pAtU6-pcoGuide1-pcoCas9-RNA-scaffold	pL1M-F5-pAtU6-pcoGuide2-pcoCas9-RNA-scaffold	pICH41800 pL1M-ELE-5
pL2B-KAN-Long35S:NLS:pcoCas9	pL2B-KAN-Long35S: NLS:pcoCas9-empty	pL1M-F4-pAtU6-pcoGuide1-pcoCas9-RNA-scaffold	pL1M-F5-pAtU6-pcoGuide2-pcoCas9-RNA-scaffold	pICH41800 pL1M-ELE-5
pL2B-KAN-Short35S:NLS:SaCas9	pL2B-KAN-Short35S: NLS:SaCas9-empty	pL1M-F4-pAtU6-SaGuide1-SaCas9-RNA-scaffold	pL1M-F5-pAtU6-SaGuide2-SaCas9-RNA-scaffold	pICH41800 pL1M-ELE-5
pL2B-KAN-Long35S:NLS:SaCas9	pL2B-KAN-Long35S: NLS:SaCas9-empty	pL1M-F4-pAtU6-SaGuide1-SaCas9-RNA-scaffold	pL1M-F5-pAtU6-SaGuide2-SaCas9-RNA-scaffold	pICH41800 pL1M-ELE-5
pL2B-KAN-RbcS2B:NLS:SaCas9	pL2B-KAN-RbcS2B: NLS:SaCas9-empty	pL1M-F4-pAtU6-SaGuide1-SaCas9-RNA-scaffold	pL1M-F5-pAtU6-SaGuide2-SaCas9-RNA-scaffold	pICH41800 pL1M-ELE-5

Vectors transformed into *B. napus* plants

Name	Backbone	Position 1	Position 2	Position 3	Position 4	Position 5	Position 6+7
pShort35S: pcoCas9	pAGM4673 pL2V	R1-pNOS-KAN-tNOS	R2-pCaMV35SShort-pcoCas9-t35S	R3-pCaMV35SShort-GFP-t35S	pL1M-F4-pAtU6-pcoGuide1-pcoCas9-RNA-scaffold	pL1M-F5-pAtU6-pcoGuide2-pcoCas9-RNA-scaffold	pICH41800 pL1M-ELE-5
pLong35S: pcoCas9	pAGM4673 pL2V	R1-pNOS-KAN-tNOS	R2-pCaMV35SLong-pcoCas9-t35S	R3-pCaMV35SLong-GFP-t35S	pL1M-F4-pAtU6-pcoGuide1-pcoCas9-RNA-scaffold	pL1M-F5-pAtU6-pcoGuide2-pcoCas9-RNA-scaffold	pICH41800 pL1M-ELE-5
pShort35S: SaCas9	pAGM4673 pL2V	R1-pNOS-KAN-tNOS	R2-pCaMV35SShort-SaCas9-t35S	R3-pCaMV35SShort-GFP-t35S	pL1M-F4-pAtU6-SaGuide1-SaCas9-RNA-scaffold	pL1M-F5-pAtU6-SaGuide2-SaCas9-RNA-scaffold	pICH41800 pL1M-ELE-5
pLong35S: SaCas9	pAGM4673 pL2V	R1-pNOS-KAN-tNOS	R2-pCaMV35SLong-SaCas9-t35S	R3-pCaMV35SLong-GFP-t35S	pL1M-F4-pAtU6-SaGuide1-SaCas9-RNA-scaffold	pL1M-F5-pAtU6-SaGuide2-SaCas9-RNA-scaffold	pICH41800 pL1M-ELE-5
pShort35S: NLS:pcoCas9	pAGM4673 pL2V	R1-pNOS-KAN-tNOS	R2-pCaMV35SShort-NLS-pcoCas9-t35S	R3-pCaMV35SShort-GFP-t35S	pL1M-F4-pAtU6-pcoGuide1-pcoCas9-RNA-scaffold	pL1M-F5-pAtU6-pcoGuide2-pcoCas9-RNA-scaffold	pICH41800 pL1M-ELE-5
pLong35S: NLS:pcoCas9	pAGM4673 pL2V	R1-pNOS-KAN-tNOS	R2-pCaMV35SLong-NLS-pcoCas9-t35S	R3-pCaMV35SLong-GFP-t35S	pL1M-F4-pAtU6-pcoGuide1-pcoCas9-RNA-scaffold	pL1M-F5-pAtU6-pcoGuide2-pcoCas9-RNA-scaffold	pICH41800 pL1M-ELE-5
pShort35S: NLS:SaCas9	pAGM4673 pL2V	R1-pNOS-KAN-tNOS	R2-pCaMV35SShort-NLS-SaCas9-t35S	R3-pCaMV35SShort-GFP-t35S	pL1M-F4-pAtU6-SaGuide1-SaCas9-RNA-scaffold	pL1M-F5-pAtU6-SaGuide2-SaCas9-RNA-scaffold	pICH41800 pL1M-ELE-5
pLong35S: NLS:SaCas9	pAGM4673 pL2V	R1-pNOS-KAN-tNOS	R2-pCaMV35SLong-NLS-SaCas9-t35S	R3-pCaMV35SLong-GFP-t35S	pL1M-F4-pAtU6-SaGuide1-SaCas9-RNA-scaffold	pL1M-F5-pAtU6-SaGuide2-SaCas9-RNA-scaffold	pICH41800 pL1M-ELE-5
pRbcS2B: NLS:SaCas9	pAGM4673 pL2V	R1-pNOS-KAN-tNOS	R2-pRbcS2B-NLS-SaCas9-t35S	R3-pCaMV35S Double-GFP-t35S	pL1M-F4-pAtU6-SaGuide1-SaCas9-RNA-scaffold	pL1M-F5-pAtU6-SaGuide2-SaCas9-RNA-scaffold	pICH41800 pL1M-ELE-5

Supplementary Table S3. Origin of plasmids.

Name	Source	Quotation
pICH51277 pL0M-P-CaMV35SShort_5UTMV	Addgene plasmid # 50268	Engler et al., 2014
pICH51288 pL0M-P-CaMV35SDouble_5UTMV	Addgene plasmid # 50269	Engler et al., 2014
pICH51266 pL0M-P-CaMV35SLong_5UTMV	Addgene plasmid # 50267	Engler et al., 2014
pICH41388 pL0M-P-CaMV35SShort	Addgene plasmid # 50253	Engler et al., 2014
pICH41373 pL0M-P-CaMV35SLong	Addgene plasmid # 50252	Engler et al., 2014
pICH44157-pL0M-P-RbcS2B	Addgene plasmid # 50258	Engler et al., 2014
pAGM5331 pL0M-5U-TMV_SP-SV40	Addgene plasmid # 50294	Engler et al., 2014
pICH41531 pL0M-SC-GFP	Addgene plasmid # 50321	Engler et al., 2014
pICH41414 pL0M-T-35S	Addgene plasmid # 50337	Engler et al., 2014
pICH47811 pL1V-R2	Addgene plasmid # 48008	Weber et al., 2011
pICH47822 pL1V-R3	Addgene plasmid # 48009	Weber et al., 2011
pICH47761 pL1V-F4	Addgene plasmid # 48003	Weber et al., 2011
pICH47772 pL1V-F5	Addgene plasmid # 48004	Weber et al., 2011
R1-pNOS-KAN-tNOS	provided by Dr. Ben Miller	Li et al., 2013
pICH54022 Dummy-2	Addgene plasmid # 48066	Weber et al., 2011
pICH49277 pL1M-ELB-3	Addgene plasmid # 48025	Weber et al., 2011
pICH41800 pL1M-ELE-5	Addgene plasmid # 48020	Weber et al., 2011
pAGM4673 pL2V	Addgene plasmid # 48014	Weber et al., 2011
pcoCas9_gene	provided by Dr. Ben Miller	Li et al., 2013
pcoCas9_RNAscaffold	provided by Dr. Ben Miller	Li et al., 2013
SaCas9_gene	provided by Dr. Holger Puchta, recloned	Wolter et al., 2018
SaCas9_RNAscaffold	provided by Dr. Holger Puchta, recloned	Wolter et al., 2018

Engler, C., Youles, M., Gruetzner, R., Ehnert, T.M., Werner, S., Jones, J.D., et al. (2014). A golden gate modular cloning toolbox for plants. *ACS Synth. Biol.* 3, 839–843. doi: 10.1021/sb4001504.

Li, J.F., Norville, J.E., Aach, J., McCormack, M., Zhang, D., Bush, J., et al. (2013). Multiplex and homologous recombination–mediated genome editing in Arabidopsis and Nicotiana benthamiana using guide RNA and Cas9. *Nat. Biotechnol.* 31, 688–691. doi: 10.1038/nbt.2654.

Weber, E., Engler, C., Gruetzner, R., Werner, S., Marillonnet, S. (2011). A Modular Cloning System for Standardized Assembly of Multigene Constructs. *PLoS ONE* 6, e16765. doi: 10.1371/journal.pone.0016765.

Wolter, F., Klemm, J., Puchta, H. (2018). Efficient in planta gene targeting in Arabidopsis using egg cell-specific expression of the Cas9 nuclease of Staphylococcus aureus. *Plant J.* 94, 735–746. doi: 10.1111/tpj.13893.

Supplementary Table S4. Supporting data for Figure 2. The table indicates the mutagenesis efficiency of each construct for each gRNA loci in *BnaA02.TAA1* (guide1–A and guide2–A) and *BnaC02.TAA1* (guide1–C and guide2–C). [a] The percentage of type of alleles per loci (WT, mutated loci) and the types of mutations (homozygote, bi-allele, heterozygote, chimera) over the total number of loci (16 – 30 independent hairy root lines per construct) is counted. [b] The percentage of type of mutated alleles over the number of all mutated loci. Homozygote: the two alleles of a locus have an identical mutation. Bi-allele: the two alleles of a locus have different mutations. Heterozygote: Only one allele is mutated. Chimera: more than two mutations per locus.

		# wt	% [a] wt	# homozygote	% [a] homozygote	% [b] homozygote	# bi-allelle	% [a] bi-allelle	% [b] bi-allelle	# heterozygote	% [a] heterozygote	% [b] heterozygote	# chimera	% [a] chimera	% [b] chimera	# total
pShort35S:pcoCas9	guide1 – A	6	37.50	1	6.25	10.00	0	0.00	0.00	4	25.00	40.00	5	31.25	50.00	16
	guide1 – C	3	18.75	5	31.25	38.46	0	0.00	0.00	5	31.25	38.46	3	18.75	23.08	16
	guide2 – A	9	56.25	0	0.00	0.00	2	12.50	28.57	3	18.75	42.86	2	12.50	28.57	16
	guide2 – C	6	37.50	0	0.00	0.00	1	6.25	10.00	7	43.75	70.00	2	12.50	20.00	16
pShort35S:SaCas9	guide1 – A	19	73.08	0	0.00	0.00	0	0.00	0.00	7	26.92	100.00	0	0.00	0.00	26
	guide1 – C	20	76.92	0	0.00	0.00	1	3.85	16.67	3	11.54	50.00	2	7.69	33.33	26
	guide2 – A	19	73.08	1	3.85	14.29	5	19.23	71.43	1	3.85	14.29	0	0.00	0.00	26
	guide2 – C	17	65.38	1	3.85	11.11	2	7.69	22.22	2	7.69	22.22	4	15.38	44.44	26
pShort35S:NLS-pcoCas9	guide1 – A	0	0.00	5	21.74	21.74	0	0.00	0.00	0	0.00	0.00	18	78.26	78.26	23
	guide1 – C	0	0.00	5	21.74	21.74	0	0.00	0.00	5	21.74	21.74	13	56.52	56.52	23
	guide2 – A	1	4.35	9	39.13	40.91	1	4.35	4.55	5	21.74	22.73	7	30.43	31.82	23
	guide2 – C	1	4.35	9	39.13	40.91	2	8.70	9.09	7	30.43	31.82	4	17.39	18.18	23
pShort35S:NLS-SaCas9	guide1 – A	12	42.86	1	3.57	6.25	7	25.00	43.75	7	25.00	43.75	1	3.57	6.25	28
	guide1 – C	13	46.43	1	3.57	6.67	5	17.86	33.33	5	17.86	33.33	4	14.29	26.67	28
	guide2 – A	14	50.00	0	0.00	0.00	6	21.43	42.86	5	17.86	35.71	3	10.71	21.43	28
	guide2 – C	12	42.86	1	3.57	6.25	1	3.57	6.25	11	39.29	68.75	3	10.71	18.75	28
pLong35S:pcoCas9	guide1 – A	3	10.00	11	36.67	40.74	0	0.00	0.00	7	23.33	25.93	9	30.00	33.33	30
	guide1 – C	5	16.67	8	26.67	32.00	1	3.33	4.00	5	16.67	20.00	11	36.67	44.00	30
	guide2 – A	11	36.67	2	6.67	10.53	3	10.00	15.79	9	30.00	47.37	5	16.67	26.32	30
	guide2 – C	11	36.67	5	16.67	26.32	0	0.00	0.00	8	26.67	42.11	6	20.00	31.58	30
pLong35S:SaCas9	guide1 – A	12	54.55	0	0.00	0.00	2	9.09	20.00	6	27.27	60.00	2	9.09	20.00	22
	guide1 – C	15	68.18	0	0.00	0.00	0	0.00	0.00	6	27.27	85.71	1	4.55	14.29	22
	guide2 – A	15	68.18	0	0.00	0.00	0	0.00	0.00	6	27.27	85.71	1	4.55	14.29	22
	guide2 – C	15	68.18	0	0.00	0.00	1	4.55	14.29	6	27.27	85.71	0	0.00	0.00	22
pLong35S:NLS-pcoCas9	guide1 – A	0	0.00	7	38.89	38.89	1	5.56	5.56	1	5.56	5.56	9	50.00	50.00	18
	guide1 – C	0	0.00	9	50.00	50.00	0	0.00	0.00	1	5.56	5.56	8	44.44	44.44	18
	guide2 – A	2	11.11	4	22.22	25.00	0	0.00	0.00	5	27.78	31.25	7	38.89	43.75	18
	guide2 – C	1	5.56	4	22.22	23.53	1	5.56	5.88	2	11.11	11.76	10	55.56	58.82	18
pLong35S:NLS-SaCas9	guide1 – A	6	26.09	0	0.00	0.00	3	13.04	17.65	7	30.43	41.18	7	30.43	41.18	23
	guide1 – C	8	34.78	0	0.00	0.00	3	13.04	20.00	6	26.09	40.00	6	26.09	40.00	23
	guide2 – A	11	47.83	1	4.35	8.33	7	30.43	58.33	1	4.35	8.33	3	13.04	25.00	23
	guide2 – C	8	34.78	1	4.35	6.67	5	21.74	33.33	5	21.74	33.33	4	17.39	26.67	23
pRBCS2B:NLS-SaCas9	guide1 – A	9	36.00	5	20.00	31.25	1	4.00	6.25	5	20.00	31.25	5	20.00	31.25	25
	guide1 – C	8	32.00	2	8.00	11.76	5	20.00	29.41	6	24.00	35.29	4	16.00	23.53	25
	guide2 – A	14	56.00	3	12.00	27.27	3	12.00	27.27	3	12.00	27.27	2	8.00	18.18	25
	guide2 – C	11	44.00	1	4.00	7.14	4	16	28.57	4	16	28.57	5	20.00	35.71	25

		# wt	% [a] wt	# homozygote	% [a] homozygote	% [b] homozygote	# bi-allelle	% [a] bi-allelle	% [b] bi-allelle	# heterozygote	% [a] heterozygote	% [b] heterozygote	# chimera	% [a] chimera	% [b] chimera	# total
SaCas9	guide1 – A	58	46.77	6	4.84	9.09	13	10.48	19.70	32	25.81	48.48	15	12.10	22.73	124
	guide1 – C	64	51.61	3	2.42	5.00	14	11.29	23.33	26	20.97	43.33	17	13.71	28.33	124
	guide2 – A	73	58.87	5	4.03	9.80	21	16.94	41.18	16	12.90	31.37	9	7.26	17.65	124
	guide2 – C	63	50.81	4	3.23	6.56	13	10.48	21.31	28	22.58	45.90	16	12.90	26.23	124
	**total**	**258**	**52.02**	**18**	**3.63**	**7.56**	**61**	**12.30**	**25.63**	**102**	**20.56**	**42.86**	**57**	**11.49**	**23.95**	**496**
pcoCas9	guide1 – A	9	10.34	24	27.59	30.77	1	1.15	1.28	12	13.79	15.38	41	47.13	52.56	87
	guide1 – C	8	9.20	27	31.03	34.18	1	1.15	1.27	16	18.39	20.25	35	40.23	44.30	87
	guide2 – A	23	26.44	15	17.24	23.44	6	6.90	9.38	22	25.29	34.38	21	24.14	32.81	87
	guide2 – C	19	21.84	18	20.69	26.47	4	4.60	5.88	24	27.59	35.29	22	25.29	32.35	87
	**total**	**59**	**16.95**	**84**	**24.14**	**29.07**	**12**	**3.45**	**4.15**	**74**	**21.26**	**25.61**	**119**	**34.20**	**41.18**	**348**
Short35S	guide1 – A	37	40.22	7	7.61	12.50	7	7.61	12.50	18	19.57	32.14	24	26.09	42.86	93
	guide1 – C	36	39.13	11	11.96	19.30	6	6.52	10.53	18	19.57	31.58	22	23.91	38.60	93
	guide2 – A	43	46.74	10	10.87	20.00	14	15.22	28.00	14	15.22	28.00	12	13.04	24.00	93
	guide2 – C	36	39.13	11	11.96	19.30	6	6.52	10.53	27	29.35	47.37	13	14.13	22.81	93
	**total**	**152**	**41.30**	**39**	**10.60**	**17.73**	**33**	**8.97**	**15.00**	**77**	**20.92**	**35.00**	**71**	**19.29**	**32.27**	**372**
Long35S	guide1 – A	21	22.34	18	19.15	25.00	6	6.38	8.33	21	22.34	29.17	27	28.72	37.50	93
	guide1 – C	28	29.79	17	18.09	26.15	4	4.26	6.15	18	19.15	27.69	26	27.66	40.00	93
	guide2 – A	39	41.49	7	7.45	12.96	10	10.64	18.52	21	22.34	38.89	16	17.02	29.63	93
	guide2 – C	35	37.23	10	10.64	17.24	7	7.45	12.07	21	22.34	36.21	20	21.28	34.48	93
	**total**	**123**	**32.71**	**52**	**13.83**	**20.88**	**27**	**7.18**	**10.84**	**81**	**21.54**	**32.53**	**89**	**23.67**	**35.74**	**372**
NLS	guide1 – A	18	19.57	13	14.13	17.57	11	11.96	14.86	15	16.30	20.27	35	38.04	47.30	92
	guide1 – C	21	22.83	15	16.30	21.13	8	8.70	11.27	17	18.48	23.94	31	33.70	43.66	92
	guide2 – A	28	30.43	14	15.22	21.88	14	15.22	21.88	16	17.39	25.00	20	21.74	31.25	92
	guide2 – C	22	23.91	15	16.30	21.43	9	9.78	12.86	25	27.17	35.71	21	22.83	30.00	92
	**total**	**89**	**24.18**	**57**	**15.49**	**20.43**	**42**	**11.41**	**15.05**	**73**	**19.84**	**26.16**	**107**	**29.08**	**38.35**	**368**
no NLS	guide1 – A	40	42.55	12	12.77	22.22	2	2.13	3.70	24	25.53	44.44	16	17.02	29.63	94
	guide1 – C	43	45.74	13	13.83	25.49	2	2.13	3.92	19	20.21	37.25	17	18.09	33.33	94
	guide2 – A	54	57.45	3	3.19	7.50	10	10.64	25.00	19	20.21	47.50	8	8.51	20.00	94
	guide2 – C	49	52.13	6	6.38	13.33	4	4.26	8.89	23	24.47	51.11	12	12.77	26.67	94
	**total**	**186**	**49.47**	**34**	**9.04**	**17.89**	**18**	**4.79**	**9.47**	**85**	**22.61**	**44.74**	**53**	**14.10**	**27.89**	**376**
NLS-pcoCas9	guide1 – A	0	0.00	12	29.27	29.27	1	2.44	2.44	1	2.44	2.44	27	65.85	65.85	41
	guide1 – C	0	0.00	14	34.15	34.15	0	0.00	0.00	6	14.63	14.63	21	51.22	51.22	41
	guide2 – A	3	7.32	13	31.71	34.21	1	2.44	2.63	10	24.39	26.32	14	34.15	36.84	41
	guide2 – C	2	4.88	13	31.71	33.33	3	7.32	7.69	9	21.95	23.08	14	34.15	35.90	41
	**total**	**5**	**3.05**	**52**	**31.71**	**32.70**	**5**	**3.05**	**3.14**	**26**	**15.85**	**16.35**	**76**	**46.34**	**47.80**	**164**
pcoCas9	guide1 – A	9	19.57	12	26.09	32.43	0	0.00	0.00	11	23.91	29.73	14	30.43	37.84	46
	guide1 – C	8	17.39	13	28.26	34.21	1	2.17	2.63	10	21.74	26.32	14	30.43	36.84	46
	guide2 – A	20	43.48	2	4.35	7.69	5	10.87	19.23	12	26.09	46.15	7	15.22	26.92	46
	guide2 – C	17	36.96	5	10.87	17.24	1	2.17	3.45	15	32.61	51.72	8	17.39	27.59	46
	**total**	**54**	**29.35**	**32**	**17.39**	**24.62**	**7**	**3.80**	**5.38**	**48**	**26.09**	**36.92**	**43**	**23.37**	**33.08**	**184**

		# wt	% [a] wt	# homozygote	% [a] homozygote	% [b] homozygote	# bi-allelle	% [a] bi-allelle	% [b] bi-allelle	# heterozygote	% [a] heterozygote	% [b] heterozygote	# chimera	% [a] chimera	% [b] chimera	# total
NLS-SaCas9 (only 35S promoters)	guide1 – A	18	23.68	1	1.32	3.03	10	13.16	30.30	14	18.42	42.42	8	10.53	24.24	51
	guide1 – C	21	27.63	1	1.32	3.33	8	10.53	26.67	11	14.47	36.67	10	13.16	33.33	51
	guide2 – A	25	32.89	1	1.32	3.85	13	17.11	50.00	6	7.89	23.08	6	7.89	23.08	51
	guide2 – C	20	26.32	2	2.63	6.45	6	7.89	19.35	16	21.05	51.61	7	9.21	22.58	51
	**total**	**84**	**27.63**	**5**	**1.64**	**4.17**	**37**	**12.17**	**30.83**	**47**	**15.46**	**39.17**	**31**	**10.20**	**25.83**	**204**
SaCas9	guide1 – A	31	64.58	0	0.00	0.00	2	4.17	11.76	13	27.08	76.47	2	4.17	11.76	48
	guide1 – C	35	72.92	0	0.00	0.00	1	2.08	7.69	9	18.75	69.23	3	6.25	23.08	48
	guide2 – A	34	70.83	1	2.08	7.14	5	10.42	35.71	7	14.58	50.00	1	2.08	7.14	48
	guide2 – C	32	66.67	1	2.08	6.25	3	6.25	18.75	8	16.67	50.00	4	8.33	25.00	48
	**total**	**132**	**68.75**	**2**	**1.04**	**3.33**	**11**	**5.73**	**18.33**	**37**	**19.27**	**61.67**	**10**	**5.21**	**16.67**	**192**

Supplementary Table S5. Stability of homozygous loci in hairy root regenerants. Detection of new mutations in regenerated plants compared to the original hairy root clone carrying pcoCas9 or SaCas9 construct. Homozygous mutated or homozygous wild type loci were studied.

	New mutations in regenerated lines (# of new mutations/# of analysed loci)				
	genome A		genome C		total
	guide1	guide2	guide1	guide2	
pcoCas9	0/6	1/4	1/7	0/5	9.1 % (2/22)
SaCas9	1/7	1/6	0/4	1/5	13.6 % (3/22)

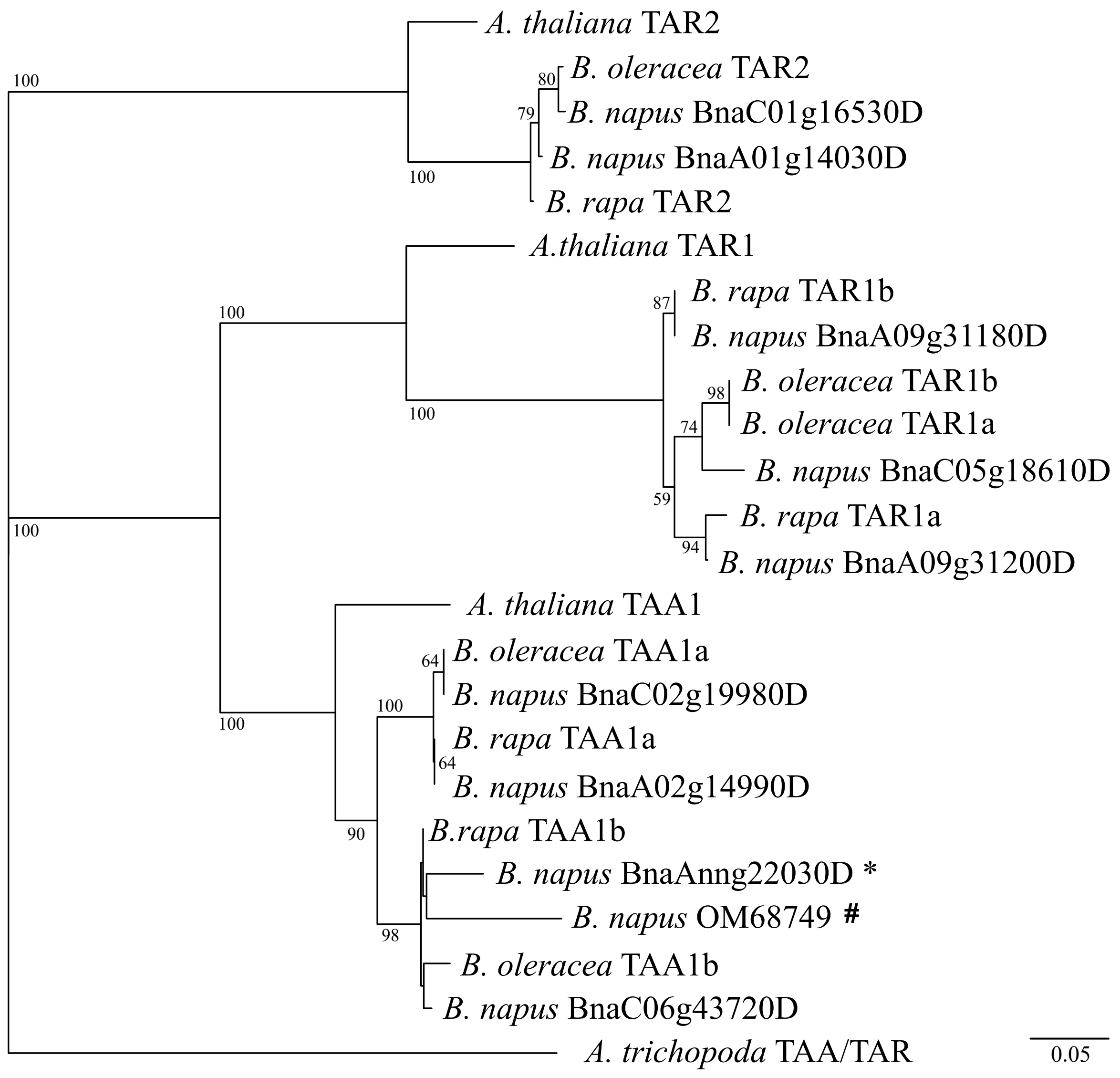

Supplementary Figure S1. Phylogenetic analysis of TAA1, TAR1, and TAR2 proteins in *Brassica napus* and related species. The neighbor-joining method was employed and resulting tree is rooted with the TAA/TAR protein of basal angiosperm *Amborella trichopoda* (GenBank, XP_011623439.1; Poulet and Kriechbaumer, 2017). Bootstrap values were calculated from 1000 replications and those equal to 50 and higher are given at each branch. The accession numbers of studied species are as follows: *Arabidopsis thaliana* (Araport; TAA1, AT1G70560.1; TAR1, AT1G23320.1; TAR2, AT4G24670.1), *Brassica oleracea* (GenBank; TAA1a, XP_013616097.1; TAA1b, XP_013590972.1; TAR1a, XP_013585148.1; TAR1b, XP_013585109.1; TAR2, XP_013618593.1), *Brassica rapa* (Phytozome; TAA1a, Brara.B01919.1.p; TAA1b, Brara.G02980.1.p; TAR1a, Brara.I03244.1.p; TAR1b, Brara.I03242.1.p; TAR2, Brara.A01473.1.p; Poulet and Kriechbaumer, 2017). The studied *Brassica napus* sequences are designated with Genoscope accessions. *, partial TAA1 protein as predicted in Genoscope. #, TAA1 protein derived from the cloned sequence (OM68749) with predicted alternative splicing.

Poulet, A., and Kriechbaumer, V. (2017). Bioinformatics analysis of phylogeny and transcription of TAA/YUC auxin biosynthetic genes. *Int. J. Mol. Sci.* 18, 1791. doi: 10.3390/ijms18081791

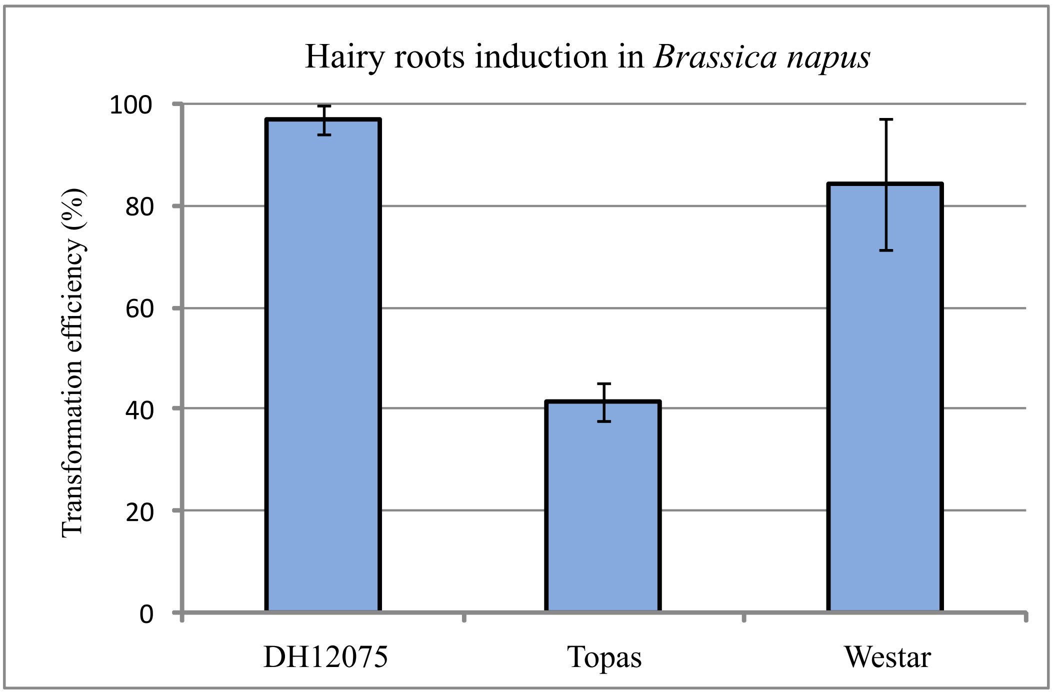

Supplementary Figure S2. Hairy roots induction in different *B. napus* cultivars. The transformation efficiency is represented as the percentage of plants with emerging hairy roots from the injected plants, assessed one month after the injection of the agrobacterial suspension (no constructs). Data were collected from three independent replicates with 18 – 24 plants per cultivar per replicate.

A

Hormone concentration	# of shoots
BAP 5 mg/L	0
NAA 5 mg/L	0
IBA 5 mg/L	0
BAP 5 mg/L, NAA 1 mg/L	13
BAP 5 mg/L, NAA 5 mg/L *	222
BAP 5 mg/L, NAA 8 mg/L	400
BAP 5 mg/L, IBA 1 mg/L	0
BAP 5 mg/L, IBA 5 mg/L	0
BAP 5 mg/L, IBA 8 mg/L	0

B

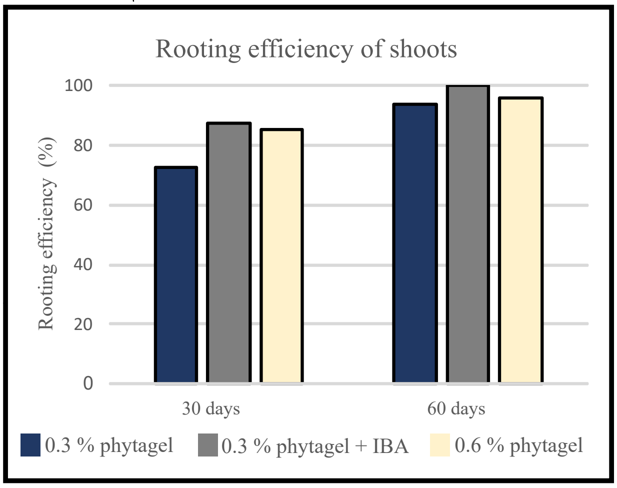

Supplementary Figure S3. Optimization of hairy roots regeneration in *B. napus* DH12075. (**A**) Effect of different concentrations of various plant growth regulators and their combination on shoot regeneration. BAP, 6-benzylaminopurine; IBA, Indole-3-butyric acid; NAA, 1-naphthaleneacetic acid.
*, concentrations of hormones used for hairy root regeneration of *Brassica spp*. by Christey and Sinclair (1992). (**B**) Impact of gelling agent (phytagel) concentration and addition of IBA (0.5 mg/L) on rooting of shoots. The percentage of shoots with emerging roots was assessed after 30 days and 60 days of cultivation on root induction medium.

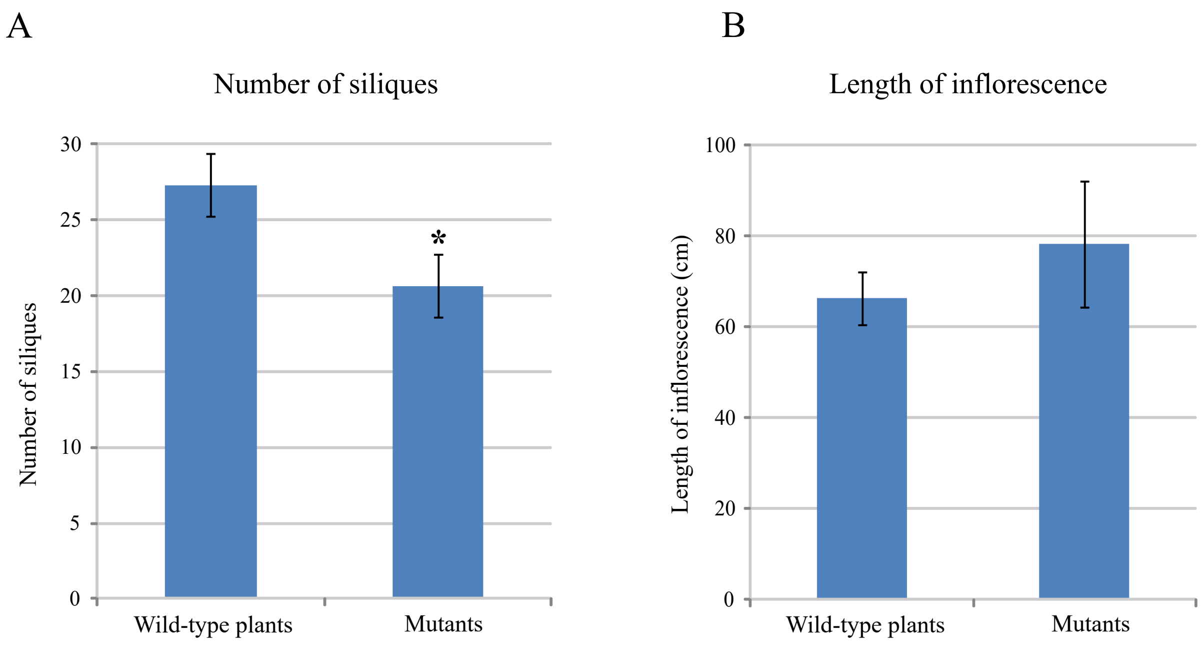

Supplementary Figure S4. Phenotypes of *BnaTAA1* double mutant primary inflorescence compared to the one of wild-type DH12075 plants. The data represent mean ± SD (n = 3). (**A**) A reduced number of developed siliques on the primary inflorescence is observed (* $p < 0.05$, Student's t-test).
(**B**) The length of the primary inflorescence between the oldest silique to the the shoot apex at mature stage does not significantly differ (Student's t-test) between control and mutant plants.

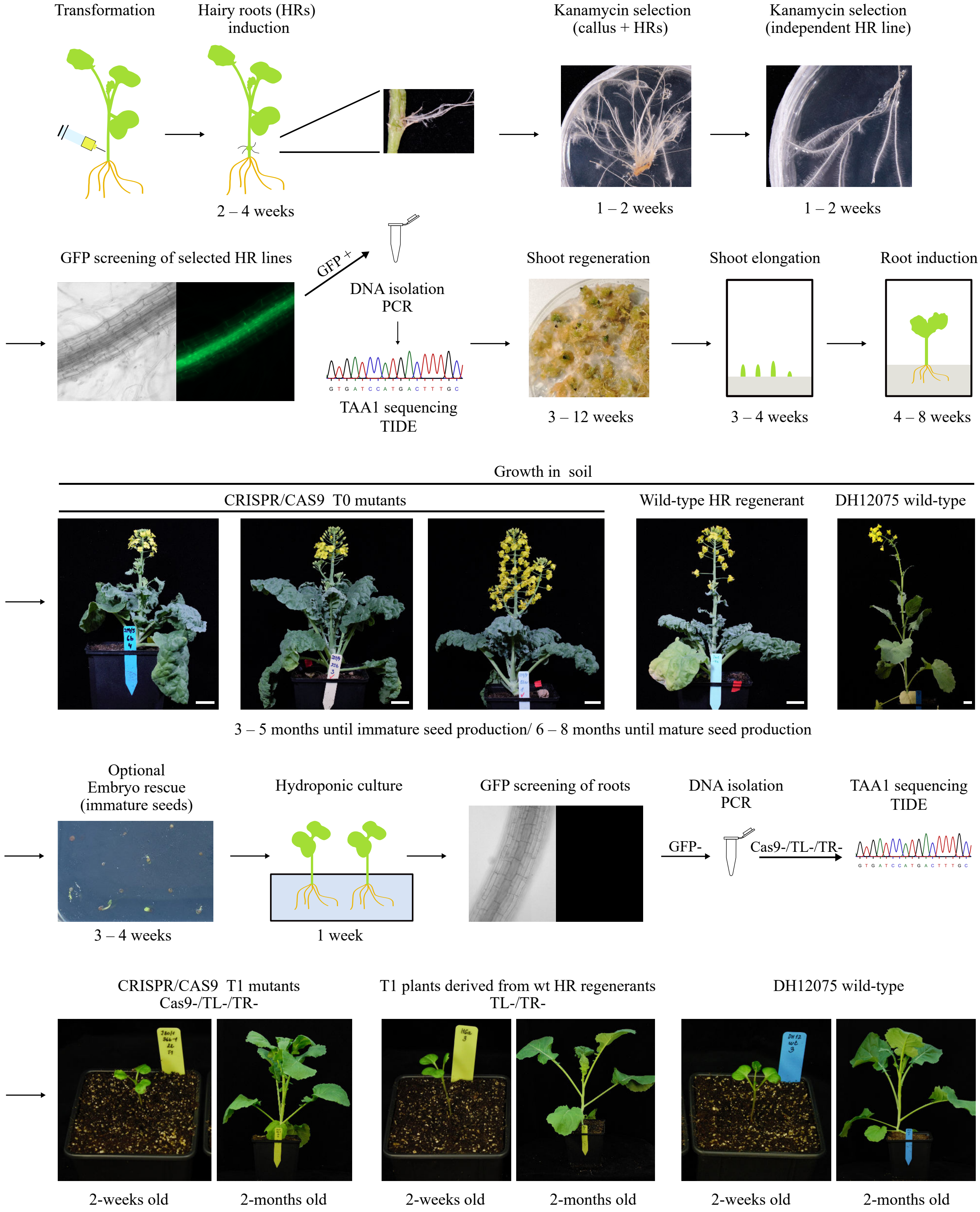

Supplementary Figure S5. Workflow scheme for transgene-free *BnaTAA1*-edited plants generation. Hairy roots (HRs) induced by injection with *Agrobacterium* carrying *Ri* plasmid and one of the CRISPR/Cas9 constructs were selected on kanamycin and screened for fluorescence signal. In GFP positive HRs, fragments of *BnaA02.TAA1* and *BnaC02.TAA1* genes were sequenced and analyzed for mutations. Selected HR lines were regenerated and transferred to the soil. To fasten the production of T1 plants, embryo rescue was performed on immature seeds. Hydroponic culture of T1 plantlets served as a convenient tool for root tip collection used for GFP screening. The GFP-negative plants were further screened for the absence of Cas9 (Cas9-) and TL-DNA (TL-) and TR-DNA (TR-) of the *Ri* plasmid. Plants negative in all features were analyzed for mutations in *BnaTAA1* genes, and their phenotypes were monitored.
